# Consumer Involvement in the Design and Development of Medication Safety Interventions or Services in Primary Care: A Scoping Review

**DOI:** 10.1111/hex.70092

**Published:** 2024-11-17

**Authors:** Megan DelDot, Esther Lau, Nicole Rayner, Jean Spinks, Fiona Kelly, Lisa Nissen

**Affiliations:** ^1^ School of Pharmacy The University of Queensland Brisbane Queensland Australia; ^2^ Centre for the Business and Economics of Health The University of Queensland Brisbane Queensland Australia; ^3^ Library, The University of Queensland Brisbane Queensland Australia; ^4^ School of Pharmacy and Medical Sciences Griffith University Gold Coast Queensland Australia

**Keywords:** consumer, consumer engagement, consumer involvement, consumer participation, medication safety, patient and public involvement, primary care

## Abstract

**Introduction:**

Medication‐related problems remain a significant burden despite the availability of various interventions and services in primary care. Involving health care consumers to design interventions or services across health disciplines is becoming more widely used as this type of engagement reportedly leads to more accessible, acceptable and sustainable health services and quality of life. We conducted a scoping review to examine when and how consumers have been involved in the design and development of medication safety interventions or services within the primary care.

**Methods:**

We searched five key databases (MEDLINE (EBSCOhost), CINAHL (EBSCOhost), PsycINFO (EBSCOhost), Embase (Elsevier) and Cochrane Library (Wiley)) for relevant articles published up to February 2024. Studies were included if they involved adult consumers (≥ 18 years), their families, carers or the wider community as stakeholders. This review only included studies where the aim was to improve safe and effective medication use, delivered exclusively in primary care. To examine consumer involvement approaches and methods we adapted a framework describing the stages of consumer involvement for the data extraction tool.

**Results:**

Overall, 15 studies were included (comprising 24 articles). Codesign, experience‐based codesign, coproduction and participatory action research were commonly used approaches. Meetings, interviews, surveys/questionnaires were commonly used methods. Two studies reported consumer involvement across all stages of the research study, and only one study described the consumer experience of being involved in the research process. The impact of consumer involvement on the effectiveness of these services or interventions was mixed.

**Conclusion:**

The potential benefits of consumer involvement in the design and development of medication safety interventions or services may not have been fully maximised, given that genuine consumer involvement across all stages of the research study appears uncommon. More transparent and consistent reporting around the description of consumers involved, their experience of being involved and overall impact and quality of consumer participation is needed.

**Patient or Public Contribution:**

This scoping review was undertaken without consumers, patients, service users, caregivers or people with lived experience or members of the public due to resource limitations. This scoping review was undertaken and written by academics, who have undertaken codesign with consumers and stakeholders and also have personal lived experience of medication‐related problems.

## 1 Introduction

Medication safety is a global health priority. In 2017, the World Health Organisation (WHO) launched its third global patient safety challenge, aiming to reduce severe, avoidable medication harm by 50% within the next 5 years [1]. A medication‐related problem (MRP) is defined as an event or circumstance involving drug therapy that actually or potentially interferes with the patient experiencing an optimum outcome of medical treatment [2]. MRPs can occur at any time during the medication process including prescribing, transcribing, dispensing, administering, monitoring and use [3, 4, 5]. Unresolved or potential medication‐related problems can lead to serious harm, yet many of these harms, with an estimate of up to 50%, including hospital admissions are considered potentially preventable [5, 6, 7]. Approximately half of medication‐related harm occurs at the prescribing/ordering stage and approximately one‐third occurs at the monitoring and reporting stages of medication use [8]. MRPs impact a consumer's quality of life, experience and interactions with the healthcare system and overall satisfaction with their care. MRPs also impose a significant financial burden on individuals, their families and the healthcare system.

Medication safety interventions or services aim to reduce the harm caused by MRPs. Some examples include electronic prescribing, clinical decision support systems, quality improvement tools for chronic diseases, electronic medication management systems, computer‐based alerts, barcoding and robotics within pharmacies, electronic health records as well as education and training programmes for health care professionals [4, 7, 9, 10]. Despite these, medication‐related problems remain a significant burden [4, 7, 9, 10]. Involving consumers may help overcome barriers to adopting interventions or services, leading to more effective and sustainable solutions for preventing medication‐related problems and harm.

Consumer involvement in health service research has shifted from being purely a participant in studies to being an active, empowered, informed partner and codesigner in the research process [5, 11]. Involvement of consumers in health care design and evaluation can lead to reduced hospital admissions [12], improved quality of life, efficiency and quality of health services [13, 14, 15, 16, 17], increased acceptance, uptake, long‐term adherence and satisfaction with health care interventions and services [15, 16, 17]. There are several important reasons for involving consumers and their lived experience in research. Firstly, consumers have a right to be involved in any research decisions that may affect their health or that of others, and researchers have a moral obligation to facilitate and ensure this involvement [18, 19, 20, 21]. Secondly, by incorporating consumers' lived experience in the research process this improves the value, quality and relevance of the research [18, 19, 20, 21]. Thirdly, engaging consumers as part of the research team increases the accountability and transparency of research [18, 19, 20, 21].

There are a number of different definitions of ‘consumers’ in the health and medical research literature. For example, the Australian Commission on Safety and Quality in Health Care Partnering with Consumers Standard defines consumers as any or all of the following: (a) a person who is receiving care in a health service organisation (b) a person who has used or may potentially use, health services or is a carer for a patient using health services or (c) a consumer representative who provides a consumer perspective, contributes consumer experiences, advocates for the interests of current and potential health service users and takes part in decision‐making processes [22]. Similarly, the Australian National Health and Medical Research Council (NHMRC) defines consumers as people who have experienced a health issue, that might receive health care or advice or otherwise use health care services [23]. For the purposes of this review the term 'consumer' will be used and encompasses both these definitions and includes patients, their friends, families, carers and members of the general public. It is acknowledged that ‘consumer’ may primarily be a term used in Australia and other jurisdictions have adopted different terms, for example, patient, public involvement and lived experienced participants.

The differing levels of consumer participation in health care research have been described as a spectrum ranging from providing balanced and objective health care information (inform) through to placing final decision‐making in the hands of the public (empower) [24]. Terminology for consumer ‘involvement’ in the design and development of health care interventions or services is confusing as it is used interchangeably with terms like ‘consultation’, ‘participation’, ‘engagement’, ‘partnership’ and ‘collaboration’ [23]. Consumer involvement is also captured in the literature under a variety of participatory methodologies, for example, ‘codesign’, ‘experience‐based codesign’, ‘coproduction’, ‘cocreation’, ‘participatory action research’, 'community‐based participatory research’ or ‘user centred design’ [15, 25, 26].

There is little known about the extent to which consumers have been involved in the design and development of medication safety interventions or services in primary care and the impact this may have. Understanding the complexity of factors associated with MRPs from a consumer perspective is key to addressing medication safety, and any interventions or services designed to prevent MRPs and potential harm should be designed with the voice and insight of the consumer in mind.

A preliminary search of PubMed, the Cochrane Database of Systematic Reviews, Open Science Framework and Johanna Briggs Institute Evidence Synthesis found no current or in‐progress scoping reviews or systematic reviews on the specific topic. The closest review identified on this topic was a scoping review that explored characteristics of medication safety programmes and outcomes to measure the effectiveness of these programmes on patient safety in primary care, however, this review did not report on consumer involvement in the design and development of these programmes [4]. The authors found that most of the medication safety programmes targeted one or more of the medication management processes and classified studies according to whether they included an organisational, professional or patient component, where the patient component focused on counselling and education [4]. The programmes ranged from complex interventions, including multidisciplinary teams, quality improvement tools, to educational packages for patients and computerised system interventions [4]. The authors concluded that more studies were required to address the various aspects of medication safety programmes and in particular, patient components [4]. Given that consumers would be the end‐users of these components it is imperative that they are involved in their design and development.

This is the first scoping review that will focus on synthesising when and how consumers have been involved in the design and development of medication safety interventions or services in primary care.

The specific review questions are:

1. What are the approach(es)/concepts to consumer involvement (e.g., codesign, co‐production, co‐creation, experience‐based codesign); **and** when have they been involved in the research process of the design and development of a medication safety intervention or service in primary care?
2. What methods or activities have been used to design and develop medication safety interventions or services with consumers (e.g. consumer advisory group, focus groups, semi‐structured interviews)?
3. Have these medication safety intervention or service studies described the consumer experience of being involved in the research process itself?
4. Have these medication safety interventions or services undergone evaluation to determine their effectiveness (e.g. health outcomes or clinical measures), including from a consumer experience perspective?

This scoping review will serve as a valuable approach for synthesising evidence about consumer involvement in medication safety intervention or service design and development in primary care [27]. This will enable the exploration of current practices, concepts, characteristics and methodologies and to identify gaps where future research may be directed [27].

## 2 Methods

Our scoping review was conducted in accordance with the Johanna Briggs Institute (JBI) methodology for scoping reviews [28] and is reported in line with the Preferred Reporting Items for Systematic Reviews and Meta‐Analyses extension for Scoping Reviews (PRISMA‐ScR) reporting guideline and checklist [29].

### 2.1 Search Strategy

The search strategy was developed by three of the authors (MD, EL, NR). An initial scoping search of PubMed and Google Scholar was undertaken to identify relevant key papers on the topic [4, 30, 31, 32, 33, 34, 35, 36, 37, 38, 39]. Three key concepts for the search (consumer, codesign and medication safety) generated a preliminary list of search terms for each concept based on clinical experience and further review of the title and abstract, search strategies and keywords used in the key papers. The search strategy was piloted in MEDLINE (EBSCOhost) and refined against known studies relevant to the research questions before conducting the full search. Five key databases were searched: MEDLINE (EBSCOhost), CINAHL (EBSCOhost), PsycINFO (EBSCOhost), Embase (Elsevier) and Cochrane Library (Wiley). Wherever possible, controlled vocabularies such as Medical Subject Headings (MeSH) were utilised alongside keywords, and then integrated using Boolean operators for comprehensive search strategies. The comprehensive search strategy for MEDLINE (EBSCOhost) is provided in Appendix 1. The initial database search ranged from inception to May 2023 and was rerun in February 2024. Title and/or abstract search limiters were applied to optimise search specificity. No restrictions were imposed on the publication language or type. The reference lists from eligible studies and systematic and scoping reviews were reviewed for further relevant studies. Trial registries were only searched within the Cochrane Library (Wiley), no relevant published studies from the registrations were identified.

### 2.2 Eligibility Criteria

#### 2.2.1 Study Design and Participants

All English language original primary research studies utilising qualitative, quantitative or mixed method study designs were included. Systematic reviews/meta‐analyses, scoping and narrative reviews, study protocols, conference abstracts, theses and dissertations, opinion pieces, editorials and non‐English language publications were excluded. The references from scoping and systematic review articles were searched but none were eligible for inclusion. Articles describing potential or theoretical interventions or services that had yet to be designed and developed were excluded. Studies were included if consumers (adults ≥ 18 years), their carers, families or the wider community were involved in the design and development of the medication safety intervention or service. We included studies where the target of the intervention or service was exclusively adults (≥ 18 years). Studies targeting towards children (< 13 years) and/or adolescents (13–17 years) were excluded.

#### 2.2.2 Interventions

We included studies that described (1) medication safety interventions or services that have been (2) developed and designed with consumers as stakeholders and (3) where the primary aim/objective was to improve the safe and effective use of medications, for example, adherence or to reduce medication adverse events, medication‐related problems and errors. We excluded interventions about illicit medicines and vaccines. Given there was no single framework that encompassed all the different terminologies describing consumer involvement we adapted and used a six‐stage codesign framework to understand when consumers were involved in the design and development of the medication safety intervention or service included in our review (Box 1) [15, 40, 41]. The first three stages (exploratory phase) gain an understanding of the consumer experience and needs, while the later three stages (development phase) focus on how to improve the experience through development and action [15, 40, 41].

Box 1: **Boyd's six stages of codesign framework [15, 40, 41]**

***Exploratory Phase***.

1. **Engage:** Proactively establishing and maintaining meaningful relationships with consumers and/or relevant stakeholders to understand and improve health care interventions or services.

2. **Plan:** Working with consumers and/or other stakeholders to come up with ideas about goals of improvement work and how to achieve them.

3**Explore:** Learning about and understanding consumer and/or stakeholders experiences of services and identifying what can be improved.

***Development Phase***.

4. **Develop:** Working with consumers and/or stakeholders to turn ideas into potential solutions (e.g., services and interventions) that will lead to better consumer experiences (intervention development).

5. **Decide:** Working with consumers and/or stakeholders to choose what improvements to make and how to make them based on further feedback.

6. **Change:** Turning improvement ideas into action. (e.g., prototype/pilot testing with end users) and finalising the intervention or service.

#### 2.2.3 Context

We included studies where the medication safety intervention or service was designed or developed for implementation exclusively in the primary care setting. For this review, primary care was defined as health care that people first seek in their community, outside of a hospital or specialist [42]. This includes general practice, Aboriginal Community Controlled Health Services, community health centres and walk‐in clinics, community pharmacies, community nursing services, mental health services, sexual and reproductive health services and allied health services [42]. We excluded hospital clinics, outpatient services or specialist services, residential aged care facilities or those developed for implementation across both primary care and hospital settings.

#### 2.2.4 Outcomes

The main outcomes of interest were the approaches/concepts and stages of the research process that involved consumers, and the methods used. This included the number and type of consumers involved, evaluation of the consumer experience of being involved in the research process and the consumer feasibility/acceptability of the intervention or service. If reported, data on the evaluation of the medication safety intervention or service effectiveness was also collected.

### 2.3 Study Selection

All identified records were collated and uploaded into Covidence systematic review software, Veritas Health Innovation, Melbourne, Australia (available at www.covidence.org.) for screening and duplicates were removed. Two reviewers (MD and EL) independently screened titles and abstracts against the inclusion criteria. The full text of potentially relevant papers was then independently assessed in detail against the inclusion criteria. Reasons for excluding full‐text papers were recorded. Any disagreements between the reviewers at any stage of the selection process were resolved through discussion and consensus or with a third reviewer.

### 2.4 Data Extraction and Charting

Data was extracted from included articles and charted using an Excel data extraction form by one author (MD), then verified by the second author (EL) (Appendix 2). If multiple articles described different parts of the design, development and evaluation process of the same intervention or service, the information was charted separately and then collated during synthesis. The following data were independently extracted from each study: author, publication year, country, title, type of medication safety intervention or service, setting and aim and outcome data (feasibility, acceptability and effectiveness). Data related to consumer involvement included framework or engagement methodology, methods of engagement, consumer characteristics (type and number), consumer involvement in research experience and consumer‐reported experience measures or outcomes were also extracted. To understand where consumers and other stakeholders were involved in the design and development of the medication safety intervention or service an adapted six‐stage codesign framework was used (Box 1) [40, 41]. If any discrepancies were identified between the extracted data, the authors met to discuss and reach a final consensus.

### 2.5 Data Synthesis

Data synthesis was completed by one author (MD) and reviewed for consistency by a second author (EL). Data were synthesised either through narrative means or quantitatively, utilising frequency counts. We described the primary care study locations and types of medication safety interventions or services.

## 3 Results

### 3.1 Study Selection

**Figure 1:**
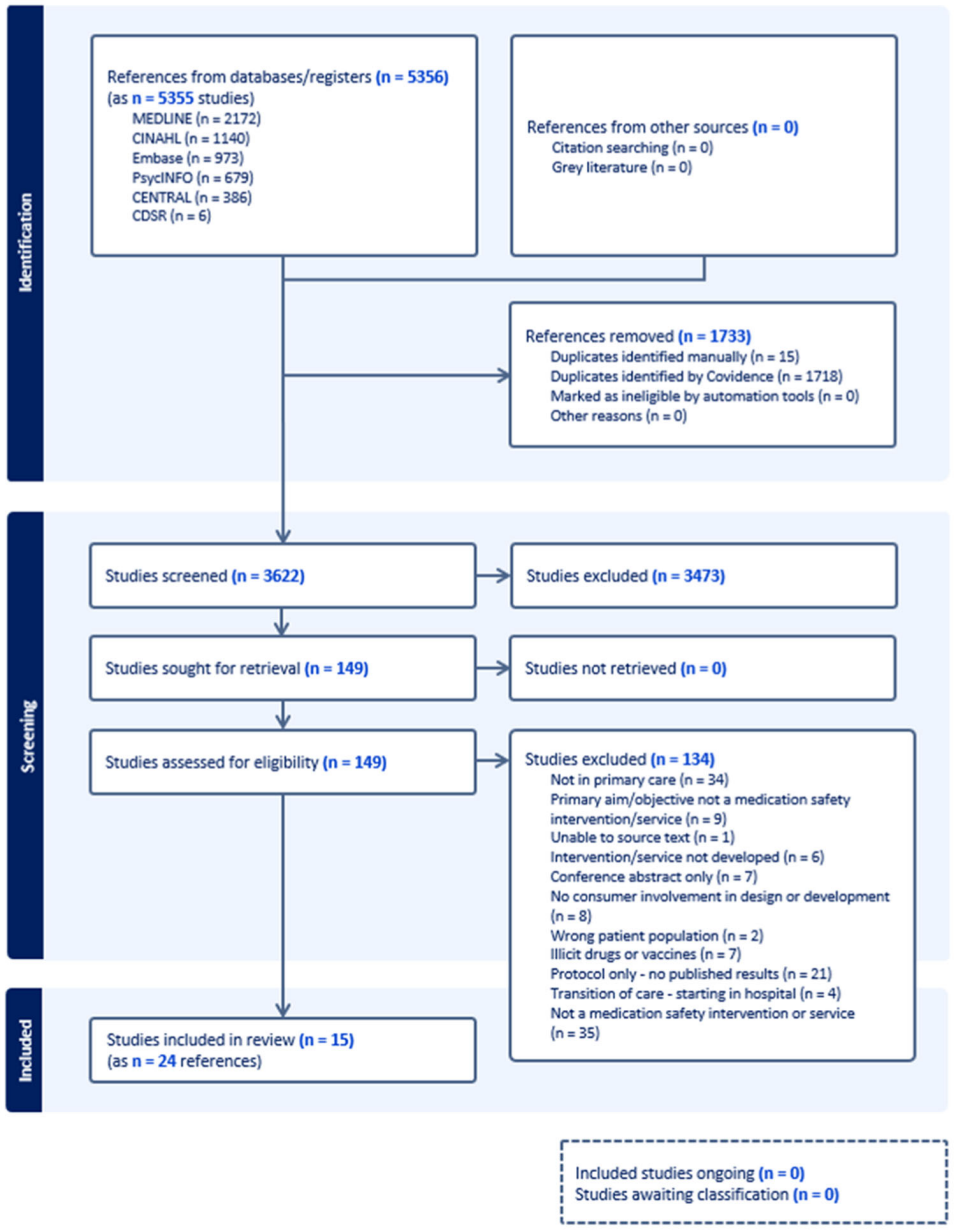
PRISMA flowchart of study selection and inclusion process.

Our search identified 5355 references. A total of 1733 duplicates were removed (15 duplicates identified manually, 1718 duplicates by Covidence). We screened 3622 in title and abstract, excluded 3473 and included 149 studies for full‐text retrieval. All records were retrieved in full text and 134 studies were excluded as they did not meet the inclusion criteria (Figure 1). Overall, 15 studies (comprising 24 references) met the eligibility criteria and were included in this final review (Figure 1 and Table 1).

**Table 1:** Summary of included studies including the type of medication safety intervention/service, evaluation of effectiveness, clinical outcomes and consumer‐reported experience measures/outcomes.

Author	Year	Country	Title	Type of medication safety intervention or service	Study setting/context	End Users of medication safety intervention or service	Aim	Outcome measures	Evaluation of intervention/service effectiveness	Consumer reported experience/outcome measures* for intervention/service
**Ben‐Zeev et al.**	2013	United States	Development and usability testing of FOCUS: a smartphone system for self‐management of schizophrenia	Mobile Health Intervention targeting: 1 medication adherence 2 mood regulation 3 sleep 4 social functioning and coping with symptoms	Community‐based rehabilitation agency	Adults living with schizophrenia	To develop a smartphone illness self‐management system for people with schizophrenia.	Mhealth intervention was developed, and patient usability testing was undertaken this revealed several intervention improvements which were incorporated into the design	Feasibility/Acceptability and preliminary effectiveness undertaken in a further study	Consumer usability sessions
Ben‐Zeev et al	2014	United States	Feasibility, Acceptability and Preliminary Efficacy of a smartphone Intervention for Schizophrenia	Mobile Health Intervention targeting: 1 medication adherence 2 mood regulation 3 sleep 4 social functioning and coping with symptoms	Community settings	Adults living with schizophrenia	To test the feasibility, acceptability, and preliminary efficacy of a smartphone intervention for schizophrenia	This study demonstrated the feasibility, acceptability, and preliminary efficacy of the FOCUS intervention for schizophrenia and introduces a new treatment model which has promise for extending the reach of evidence‐based care beyond the confines of a physical clinic using widely available technologies.	FOCUS intervention was helpful in reduction of positive symptoms of schizophrenia (PANSS‐positive scale), general symptoms of psychopathology (PANSS general psychopathology scale), and depression (BDI‐2) over the 1‐month trial. There were no significant changes in sleep or beliefs about medications.	Approximately 90% of participants rated the intervention highly acceptable and usable. They reported feeling very confident, comfortable, and satisfied using the intervention.
**Breslin et al.**	2008	United States	The design of a decision aid about diabetes medications for use during the consultation with patients with type 2 diabetes	Clinical Decision Aid (DA)	Primary care	Adults living with diabetes	To describe the process used to develop a medication choice decision aid (DA) for patients with type 2 diabetes.	A novel interactive decision aid was designed ‐ future study on impact on HbA1c levels and medication adherence	Prototype used in actual clinical encounters Prototype shared with CAG	Not described
Mullan et al.	2009	United States	The diabetes mellitus medication choice decision aid: a randomised trial	Clinical Decision Aid	Primary care and family medicine sites	Adults living with diabetes	To undertake a pilot, cluster randomised trial	Patient acceptability Patient involvement in decision making Clinician survey regarding tool's helpfulness and appropriateness Adherence assessment at 1,3,6 months Haemoglobin A1c levels at 6 months	No significant impact on HbA1c Improved knowledge, No better adherence and persistence	Patients receiving DA found the tool more helpful, improved knowledge, more involvement in decisions about diabetes medicines
**Cedillo et al.**	2022	United States	Towards Safer Opioid Prescribing in HIV care (TOWER): a mixed‐methods, cluster randomised trial	Multi‐faceted intervention: 1 Patient facing opioid management App 2 Progress note template 3 Primary care provider training	Primary care clinics	Adults living with HIV, Healthcare practitioners	To develop and test a CDC Guideline implementation strategy (TOWER)	TOWER intervention was developed and evaluated. Clinical guideline adherence Patient centred measures: pain intensity and function, mood, substance use, medication use and adherence, relationship with provider, stigma, and discrimination	Primary care physicians (PCPs) randomised to TOWER were 48% more adherent to guidelines with significant improvements in use of: ‐ non pharmacologic treatment ‐ functional treatment goals ‐ opioid agreements ‐ prescription drug monitoring programmes (PDMPs) ‐ opioid benefit/harm assessment ‐ naloxone prescribing High level of confidence in conducting care processes. There was no worsening of patient reported outcome measures.	PROM ‐ No evidence of intervention associated change in any of the patient centred measures used.
Robinson‐Papp et al.	2019	United States	Decreasing risk among HIV patients on opioid therapy for chronic pain: Development of the TOWER intervention for HIV care providers	Multi‐faceted intervention with a patient facing opioid management app, prescriber training, progress note template	Primary care	Adults living with HIV, Healthcare practitioners	To develop and do preliminary testing of a theory‐based intervention, called TOWER (TOW ard Saf ER Opioid Prescribing), designed to support HIV primary care providers in Centre for Disease Control Guideline ‐ adherent opioid prescribing practices with people with HIV (PWH) who are already prescribed opioids for chronic pain.	TOWER intervention to improve adherence was developed.	Future study to demonstrate efficacy and feasibility and the ability of TOWER to improve outcomes in PWH	Not described ‐ future study
**Deb et al.**	2021	United Kingdom	Short‐Term Psychoeducation for Caregivers to Reduce Overmedication of People with Intellectual Disabilities (PwID) (SPECTROM): Development and Field Testing	Complex intervention ‐ Training program for support staff − 14 modules, resources, face to face training	Community Homes	Support staff and caregivers	To develop a training program, SPECTROM, for support staff to help reduce overmedication in people with intellectual disability, carry out field testing and conduct a process evaluation to gather feedback from the participants on the training to assess implementation issues.	A training program was developed. Outcome data − 2 questionnaires (before and after training) + interviews about acceptability, applicability, practicality, and relevance Assessment of knowledge of psychotropic medication use in PwID and to assess participants' change in attitude toward challenging behaviour and the person behind the behaviour (Management of Aggression and Violence Scale Revised Intellectual Disabilities MAVAS‐R‐ID).	MAVAS‐R‐ID scores showed change in staff attitude to ‘medication management’ domain was statistically significant (*p* < 0.05). Psychotropic knowledge questionnaire showed statistically significant post‐training improvement in correct responses (*p* < 0.05). Spectrum is a useful training program that helps to change support staff's attitude toward challenging behaviours and improve their knowledge of psychotropic drugs.	Not described
Deb et al.	2020	United Kingdom	Short‐term Psychoeducation for Carers to Reduce Overmedication of People with Intellectual Disabilities (PwID) (SPECTROM): Study Protocol	Training program − 14 modules, resources, face‐to‐face training	Community Homes	Support staff and caregivers	To develop a psychoeducational programme (PEP) for care staff	Study protocol only	Study protocol only	Study protocol only
**Desai et al.**	2023	United States	Identifying barriers and facilitators along the hepatitis C care cascade to inform human‐centred design of contextualised treatment protocols for vulnerable populations in Austin, Texas: a qualitative study	Treatment protocols	Primary care clinics	Healthcare practitioners	To identify contextual barriers and facilitators to treatment of hepatitis C at a local community health centre	The specific needs of people who inject drugs and people experiencing homelessness patient populations informed barriers and facilitators of HCV care and were incorporated into the development of contextualised treatment protocols	Not described ‐ future study	Not described
**Elliott et al.**	2017	Australia	Development of a clinical pharmacy model within an Australian home nursing service using co‐creation and participatory action research: the Visiting Pharmacist (ViP) study	Clinical pharmacy support service for community nurses	Home‐based care (community)	Community nursing staff	To develop a collaborative, person‐centred model of clinical pharmacy support for community nurses and their medication management clients	A clinical pharmacy model of care was developed within a home nursing service. Feedback and reflections from minutes, notes and transcripts from project team meetings, clinical pharmacists' reflective diaries, and interviews, meetings with clinical nurses, reference group meetings and interviews and focus groups with older people, carers, nurses, general practitioners, and community pharmacists.	Model of care allowed nurses to refer directly to the pharmacist, enabling timely resolution of medication issues	Clients and carers reported (in depth interviews/focus groups) that pharmacist visits provided an opportunity for them to ask questions, sometimes simplification of the medication regimen occurred saving the clients' money, reducing the need for nurse visits, reduced side effects. Feedback sought indicated model was well accepted.
Lee et al.	2018	Australia	Improving medication safety for home nursing clients: A prospective observational study of a novel clinical pharmacy service−The Visiting Pharmacist (ViP) study	Clinical pharmacy support service for community nurses	Home‐based care	Community nursing staff	To explore the number and type of medication‐related problems (MRPs) and medication treatment authorisation (medication order) discrepancies identified and addressed by clinical pharmacists	Evaluation and review of client's medicines data, including treatment authorisations and pharmacist medication review reports: Prevalence and type of pharmacist identified medication‐related problems. Prescribers' uptake of pharmacist recommendations to address MRPs. Accuracy of medication treatment authorisations. Number of clients who received a new, updated treatment authorization as a result of pharmacist review.	Eighty‐four clients (median 86 years, 6 health conditions, 13 medications) were reviewed. The pharmacists identified 334 MRPs (median 4 per client) and 307 medication discrepancies in treatment authorisations (median 2 per client). The pharmacists made 282 recommendations to prescribers to address MRPs; 148 (52.5%) recommendations were acted on, resulting in 190 medication changes for 60 (71.4%) clients (median 2 per client). The pharmacists prepared, or assisted GPs to update, treatment authorisations for 68 (81%) clients. Integrating pharmacists into a home nursing service identified and addressed MRPs and medication treatment authorization discrepancies, hence contributing to enhanced medication safety.	Not described
**Hanlon et al.**	2021	Ireland	Designing an e‐learning tool to support health practitioners caring for patients taking multiple medications	Medication adherence e‐learning tool/resource to help general practitioners and nurses support medication adherence	General practice	Healthcare practitioners	To develop a patient‐informed e‐learning resource to help GPs and nurses to support long‐term medication taking in multimorbidity	Development of a flexible eLearning tool for continuous professional development that has been integrated into general practice and clinical education programmes as a supportive tool.	Not described ‐ future study	Not described
**Holmqvist et al.**	2023	Sweden	Older Persons' and Health Care Professionals' Design Choices when Co‐designing a Medication Plan Aiming to Promote Patient Safety: Case Study	Medication Plan Prototype in electronic health record	Regional Home‐Based care	Older persons	To identify participants' needs and requirements for a medication plan and explore their reasoning for different design choices	An iteratively developed medication plan linked with a medication list was developed with an existing electronic health record.	Not described ‐ Future study to test prototype in clinical practice	Not described ‐ future study
Holmqvist et al	2023	Sweden	How Older Persons and Health Care Professionals Co‐designed a Medication Plan Prototype Remotely to Promote Patient Safety: Case Study	Medication Plan Prototype in electronic health record	Community‐dwelling	Older persons	To describe how remote Codesign was applied to create a medication plan prototype and to explore participants' experiences with this approach.	Participants' experiences with codesign approach	Not described ‐ Future study to test prototype in clinical practice	Not described ‐ future study
Holmqvist et al.	2019	Sweden	Older persons' experiences regarding evaluation of their medication treatment ‐ An interview study in Sweden	Evaluation of medication safety concerns	Community dwelling	Older persons	To identify opportunities to make the medication use process safer	Analysis of older persons' experiences	Older persons want to be actively involved in their medication evaluations, and this may represent an underutilized resource in the pursuit of patient safety. Their trust in physicians to undertake evaluations on a regular basis, although that does not necessarily occur, may cause harm. Patient safety could benefit from a Coproduction approach to medication evaluations, with health‐care professionals explicitly sharing information with older persons and agreeing on responsibilities related to on‐going medication treatment.	Not described ‐ Future study
**Kassavou et al.**	2019	United Kingdom	Development and piloting of a highly tailored digital intervention to support adherence to antihypertensive medications as an adjunct to primary care consultations	Digital medication adherence intervention ‐ text and voice messaging	Primary care setting	Adults with hypertension	To describe the development and piloting of a highly tailored text and voice message intervention to support adherence to antihypertensive medication	A 1‐month pilot intervention was conducted to assess (a) implementation procedures; (b) uptake, retention, fidelity, and engagement with the intervention; (c) participants' views, understanding and actions on the intervention content; and obtain (d) recommendations for improvement.	During the 1‐month intervention, an average of 29 messages were scheduled, of which 22.52 were received by patients. On average, 37 calls were made, of which 2.8 failed to be made due to technical issues and 13 calls were made but failed to go through because they were not picked up by the participants. On average, three calls were repeated and received by participants. Also, 10 out of the 17 participants had chosen the calls to be repeated if not answered, and 5 out of the 17 participants had provided a secondary number for the calls to be repeated, if there was no answered at the primary number. Tailored automated text and voice message interventions are feasible ways to improve medication adherence as an adjunct to primary care.	Overall, patients were engaged with the intervention and made inbound calls to change the schedule of the messages or report about intervention content. Participants suggested that the intervention was acceptable and easy to use, and they provided their views about specific features of the intervention delivery mode and intervention content. Specifically, the tailored schedule of the messages, the personalisation and the variation of the content were found to be particularly appealing and were perceived to promote engagement with the intervention
Kassavou et al.	2020	United Kingdom	A Highly Tailored Text and Voice Messaging Intervention to Improve Medication Adherence in Patients with Either or Both Hypertension and Type 2 Diabetes in a UK Primary Care Setting: Feasibility Randomised Controlled Trial of Clinical Effectiveness	Digital medication adherence intervention ‐ text and voice messaging	Primary care setting	Adults with hypertension	To assess if patients with either or both hypertension and type 2 diabetes, who used the intervention for 3 months in addition to usual care had improved medication adherence and if they differed in terms of blood pressure or haemoglobin A1c and quality of life.	Behavioural efficacy Recruitment and attrition rate Intervention fidelity, engagement and satisfaction Efficacy to support medication adherences and feasibility of clinical effectiveness at 3 months (T2) (using 2 self‐reported items of adherence)/event monitoring system caps for seven days after T2. Blood pressure and haemoglobin A1c at T2. Quality of life measure using 5 level EQ‐5D.	Medication adherence was significantly improved in the intervention group compared with the control group (t116 = 2.27; *p* = 0.02, 2‐tailed). Systolic blood pressure was 0.6 mmHg (95% CI − 7.423 to 6.301), and haemoglobin A1c was 4.5 mmol/mol (95% CI − 13.099 to 4.710) lower in the intervention group compared with the control group. Attrition rate was low. Intervention fidelity was 100% for total duration.	Patients reported the intervention was easy to use and were satisfied with the experience with the intervention.
**Latif et al.**	2019	United Kingdom	Supporting the provision of pharmacy medication reviews to marginalised (medically underserved) groups: a before/after questionnaire study investigating the impact of a patient‐professional co‐produced digital education intervention	Online Digital Education Intervention: 1. Discovering underserved communities 2. Exploring medicine experiences 3. Effectively interacting and engaging with patients	Community Pharmacy	Pharmacy staff	To develop an online digital educational intervention to improve pharmacy staffs' intention to offer a community pharmacy medication review service to medically underserved groups	The primary outcome measure was ‘behaviour change intention’ using a validated 12‐item survey measure. The secondary outcome measure was pharmacist self‐reported recruitment of underserved groups to the medication review service.	A before/after comparison analysis found an improving trend in all the five constructs of behaviour change intention (intention, social influence, beliefs about capabilities, moral norms and beliefs about consequences), with a significant increase in mean score of participants' ‘beliefs about capabilities' (0.44; 95% CI 0.11–0.76, *p* = 0.009). In the short term, no significant change was detected in the number of patients being offered and the patient completing a medication review.	Not described
Latif et al	2018	United Kingdom	Giving Voice to the Medically Under‐Served: A Qualitative Coproduction Approach to Explore Patient Medicine Experiences and Improve Services to Marginalised Communities	Online Digital education intervention for community pharmacy	Community Pharmacy	Pharmacy staff	This study forms part of a wider project to codevelop and evaluate a digital educational intervention for community pharmacy. The aim of this paper is to explore the medicine needs of patients from marginalised communities and suggest practical way on how services could be better tailored to their requirements.	Qualitative data from semi‐structured interviews and workshops to inform intervention development. Develop outline for the contents of e‐learning and written specification.	Future study	Findings revealed that patients from marginalised communities reported poor management of their medical conditions and significant problems with adherence to prescribed medicines. Their experience of pharmacy services was found to be variable with many experiencing discrimination or disadvantage as a result of their status.
**Lynch et al.**	2022	Ireland	Supporting safe and gradual reduction of long‐term benzodiazepine receptor agonist (BZRA) use: Development of the SAFEGUARDING‐BZRAs toolkit using a codesign approach	Toolkit (24 behaviour change techniques)	Primary care	Healthcare practitioners	To develop an intervention to support discontinuation of long‐term BZRA use among willing individuals	Development of a toolkit using a systematic and theory‐based approach that addresses identified limitations of previous research. Future study to assess usability and feasibility and potential to effectively support safe and gradual reduction of long‐term BZRA use	Not described – future study	Not described
**Pepper et al.**	2023	South Africa	“If I don't take my treatment, I will die and who will take care of my child?”: An investigation into an inclusive community‐led approach to addressing barriers to HIV treatment adherence by postpartum women living with HIV	Community‐led intervention or programme: 1. Creation of life plans 2. Discussion of HIV stigma in local schools, health facilities and community organisations 3. Life skills training and mentoring 4. Peer support through WhatsApp groups	Rural Community	Post Partum Women living with HIV and their communities	To demonstrate the strength of an inclusive research and programme approach to improving the lives of people living with HIV and simultaneously Antiretroviral (ARV) adherence	Two main barriers to ARV adherence emerged: the anticipated stigma associated with issues of disclosure and poverty epitomised by alcohol abuse, gender‐based violence and hunger. A programme of support for all women living with HIV in the area was developed that addressed each of the issues raised by the co‐researchers and is run via a community‐led process where the participants lead on design, implementation, and monitoring and ultimately will revise the programme as needed.	Not described ‐ future study	Not described
**Perngmark et al.**	2022	Thailand	Family Participation to Promote Medication Adherence Among Thai‐Muslim Older Adults with Hypertension: Action Research Study	Multi‐faceted Intervention program: 1. Assessment and screening 2. Implementation ‐ health education, medication workshop 3. Phone‐call visits 4. Home visit 5. Follow‐up and evaluation	Community Health Centre	Thai‐Muslim older adults	To develop, implement and evaluate an Islamic‐based intervention program to promote medication adherence among Muslim older adults with uncontrolled hypertension in southern Thailand	Developed an intervention Measure of Blood Pressure levels, drug adherence scores, residual pill counts report, family participation score. Semi‐structured interviews about program feasibility, acceptability, adequacy, and satisfaction.	Core program outcomes obtained with BP readings returning to normal levels, high drug adherence scores, low residual pill counts.	Family dyads reported improved knowledge and awareness of drug use and drug side effects, self confidence in handling drug side effects, reported a clearer understanding of caregiver's roles. Family/caregivers felt better prepared to assist with medication adherence
**Remien et al.**	2013	South Africa	Masivukeni: development of a multimedia based antiretroviral therapy (ART) adherence intervention for counsellors and patients in South Africa	Multimedia‐Based Antiretroviral Therapy Adherence Intervention ‐ online interactive modules for patients/lay counsellors	Healthcare Clinic	Adults living with HIV, Lay counsellors	To develop an intervention that could be used by lay counsellors to help people living with HIV in resource‐limited settings achieve and maintain high levels of ART adherence	Intervention adapted and the overall experience of the intervention (acceptability) and feasibility in South African context examined.	27 patients initiated the intervention, 85% completed at least four of the six sessions. Counsellors said the framework increased their ability to do their job, empowered them to address barriers and facilitators of adherence. Videos helped patients engage more with the counsellors. Counsellors indicated that sessions 5 and 6 were too long and repetitive. Clinic physicians, nurses and the pharmacist reported positive changes in patients, particularly in consistent clinic attendance.	Most patients found the sessions engaging and reported a better understanding of difficult concepts related to treatment and health including how ART works, viral resistance, and dangers of alcohol. High acceptance and feasibility of intervention.
**Silcock et al.**	2023	United Kingdom	Co‐designing an intervention to improve the process of deprescribing for older people living with frailty in the United Kingdom	Deprescribing intervention	Primary care	Older people living with frailty, Healthcare practitioners	To Codesign an intervention, supported by a logic model to increase the engagement of older people living with frailty in the process of deprescribing	Priorities for improving the current deprescribing process were successfully identified, solutions developed and structured as a complex intervention. The complex intervention responded to priorities about clarity for older people about what was happening at all stages in the deprescribing process and the quality of one‐to‐one consultations. Further work is underway to complete prototyping of the intervention and conduct feasibility testing.	Not described ‐ further work is undergoing to conduct feasibility testing with primary care partners before full evaluation	Not described

*Note:* **Bolded** author names represent the original 15 studies included in the review.

* These are commonly reported as Patient Reported Experience Measures (PREMs) or Patient Reported Outcome Measures (PROMs).

There were a mixture of study types in this review with six utilising a mixed method approach [43, 44, 45, 46, 47, 48], and nine were qualitative studies [36, 49, 50, 51, 52, 53, 54, 55, 56]. Studies were from a range of countries, with the majority coming from the United Kingdom (*n* = 4), the United States (*n* = 4), Ireland (*n* = 2), South Africa (*n* = 2), Australia (n = 1), Sweden (*n* = 1) and Thailand (*n* = 1). The included studies were published between 2008 and 2023 with 11 of the studies published in the last 6 years. Interventions or services were developed for use within a variety of primary care settings including a community‐based rehabilitation agency [43], primary care clinics [44, 50, 55], patients' homes, [45, 46, 51] general practice [52], community pharmacy [48], a rural community [54] and a community health centre [56]. Some studies did not provide specific details about the primary care facility just that the study was undertaken in the primary care setting [36, 47, 49, 53].

### 3.2 Types of Medication Safety Interventions or Services

The types of medication safety interventions or services varied and targeted different end users and different parts of the medication process (Table 1). Two mobile health interventions were developed to assist patients with medication adherence and one study aimed to improve patient safety through the development of a medication plan [43, 46, 47]. Four studies focused on prescribing or deprescribing and shared decision‐making to minimise medication‐related problems [36, 49, 50, 53]. Four studies focused on training and education of health care professionals, caregivers, lay counsellors and patients to improve their knowledge and skills [45, 48, 52, 55]. Three studies described multi‐faceted approaches to medication safety [44, 54, 56].

### 3.3 Consumer Involvement Approaches

#### 3.3.1 Approaches, Concepts and Methods for Consumer Involvement

Different consumer involvement approaches were described by the studies, with codesign (*n* = 4 studies), coproduction *n * = 2 studies) participatory action research (PAR) (*n* = 2 studies), Patient and Public Involvement/Engagement (PPI/PPE) ((*n* = 2 studies) and experience‐based codesign (*n* = 2 studies) being the most common types (Table 2). Other reported approaches (*n* = 1 of each) were cocreation, user‐centred design, design research, public deliberation, human‐centred design and community‐based participatory research. Methods of consumer involvement varied across the studies with interviews (*n* = 12 studies), questionnaires/surveys (*n* = 8 studies), workshops (*n* = 5 studies), meetings (*n* = 5 studies) and focus groups (*n* = 4 studies) commonly described (Table 2). Other methods of involvement described across the studies were direct or think‐aloud observation sessions (*n* = 2 studies), consumer advisory groups/panel meetings (*n* = 3 studies), codesign event (*n* = 1 study), reflective diaries (*n* = 1 study), photovoice (*n* = 1) and public deliberations session (*n* = 1 study) (Table 2). There was no apparent link between the consumer involvement approach/methodology and methods/activities used.

**Table 2:** Consumer involvement characteristics, approaches and methods in included studies.

Author	Consumer involvement approach/methodology	Methods/Activities of consumer involvement	Types of consumer involvement	Number of consumers	Engage	Plan	Explore	Develop	Decide	Change	Evaluation of consumer involvement in design/development of intervention or service
**Overall summary of where consumer involvement was reported across the research process (n** = **15 studies)**					**8**	**8**	**12**	**14**	**8**	**8**	Not applicable
**Ben‐Zeev et al.**	User‐centred design Mixed methods	Survey, Group discussion, Think aloud observations, questionnaire	Schizophrenia or schizoaffective disorder “think aloud” session + Questionnaire	904 (stage 1) 12 (stage 3)	✓ Yes C/I	✗	✓ Yes C/I	✓ Yes C/I	✓	✓ Yes C/I	Not described
Ben‐Zeev et al.	User‐centred design	Clinical staff identified individuals who were viable candidates for the study	Patients with schizophrenia or schizoaffective disorder	33	✗	✗	✗	✗	✗	✗	Not described
**Breslin et al.**	Design Research Approach, Qualitative	Patient Advisory Group Meetings, Direct observation	Patient Advisory Group Patients	8 25	✓ Yes C/I	✓ Yes C/I	✓ Yes C/I	✓ Yes C/I	✓ Yes C/I	✓ Yes C/I	Not described
Mullan et al.	Randomised controlled trial Mixed methods	Survey, pictorial instrument, visit video recordings, interviews	Patients with type 2 diabetes	48	n/a	n/a	n/a	n/a	n/a	n/a	Not described
**Cedillo et al.**	Cluster randomised trial Information, Motivation, Behavioural skills model of behaviour change Mixed methods	Questionnaire	Patients	40	✓ Yes C/I	✓ Yes C/I	✗	✗	✗	✓ Yes C/I	Not described
Robinson‐Papp et al.	Public deliberation	Interviews, Public deliberation sessions	People with HIV (PWH)	43	✓	✗	✓ Yes C/I	✓ Yes C/I	✓	✓ Yes C/I	Not described
**Deb et al**	Experience Based Codesign (EBCD), Coproduction Medical Research Council's guidelines (MRC) Mixed methods	Focus groups, codesign event, Learning disability advisory group, Pep Development Group meetings, semi‐structured interviews, questionnaires	Family carers Adults with intellectual disabilities and their families	5 Not stated	✗	✗	✓	✓ Yes C/I	✓ Yes C/I	✓	Not described
Deb et al.	Coproduction	Focus groups, codesign event, Learning disability advisory group, Pep Development Group meetings, semi‐structured interviews, questionnaires	Adults with intellectual disabilities and their families	Not stated	✓ Yes C/I	✓ Yes C/I	✗	✗	✗	✗	Study protocol only
**Desai et al.**	Human‐centred design Practical, Robust Implementation and Sustainability model (PRISM) framework Qualitative	Semi‐structured interviews, design workshops	Patients with HCV	10	✗	✗	✓ Yes C/I	✓	✓	✗	Not described
**Elliott et al.**	Cocreation, Codesign, Participatory Action Research Qualitative	Project team meetings, stakeholder reference group meeting, reflective diaries, interviews, focus groups, case notes, community nurse clinical meeting, records of direct communication with research team	Older people Carers	27 18	✓ Yes C/I	✓ Yes C/I	✓ Yes C/I	✓ Yes C/I	✓ Yes C/I	✓	Not described
Lee et al.	Prospective observational study	Review of clients' medication	Clients	84	n/a	n/a	n/a	n/a	n/a	n/a	Not described
**Hanlon et al.**	Patient and Public Involvement (PPI), Collective intelligence Qualitative	Collective intelligence workshop, scenario‐based design	Patient Representatives	4	✗	✗	✓	✓ Yes C/I	✓	✗	Not described
**Holmqvist et al.**	Codesign Double Diamond Framework, Case study design Mixed methods	Survey, web‐based workshop, interviews	Older persons	5	✗	✗	✗	✓ Yes C/I	✓ Yes C/I	✓ Yes C/I	Described in another article
Holmqvist et al.	Codesign Design Council Double Diamond Framework	Workshops, survey	Older persons	5	✗	✗	✗	✓ Yes C/I	✓ Yes C/I	✓ Yes C/I	The experiences of a remote codesign initiative with 14 participants in a regional health care system in southern Sweden were explored ‐ questionnaire and web‐based workshop. Participants rated the experiences of the codesign initiative very high. In addition, the balance between how much involved persons expressed their wishes and were listened to was considered very good. The remote codesign process seemed appealing, by balancing opportunities and challenges and building an inviting, creative, and tolerant environment.
Holmqvist et al.	Consumer interviews ‐ qualitative Medication use model	Semi‐structured interviews	Older persons	20	✗	✗	✓ Yes C/I	✗	✗	✗	Not described ‐ future study
**Kassavou et al.**	Patient and Public Involvement/Engagement (PPI/PPE) Medical Research Council (MRC) Framework Mixed methods	Interviews, think‐aloud sessions, focus groups, consultations, questionnaire	Patients interviews PPI Events Interviews Meetings Focus groups Experiential focus groups PPI consultations Patients PPI members	19 100 13 2 12 6 12 2 2	✗	✗	✓ Yes C/I	✓ Yes C/I	✓	✓ Yes C/I	Not described
Kassavou et al.	Randomised controlled trial	Questionnaire, self‐reports, digital log files	Nonadherent patients	135	n/a	n/a	n/a	n/a	n/a	n/a	Not described
**Latif et al.**	Coproduction Mixed Methods	Semi‐structured interviews, Advisory panel, workshops, and survey	Patient support groups Advisory panel	3 2	✓ Yes C/I	✓ Yes C/I	✗	✗	✗	✓	Not described
Latif et al.	Coproduction	Workshops, semi‐structured interviews	Patients at eLearning development workshops Review workshops patients Interviews	19 14 10	✗	✗	✓ Yes C/I	✓ Yes C/I	✓ Yes C/I	✗	Not described
**Lynch et al.**	Codesign UK Medical Council Complex Intervention Framework, Theoretical Domains Framework (TDF) Qualitative	Interviews, codesign team meetings	Patients with current/previous experience Experts by experience	28 5	✗	✗	✓ Yes C/I	✓ Yes C/I	✓ Yes C/I	✗	Not described
**Pepper et al.**	Participatory Action Research (PAR) Qualitative	Photovoice (visual participatory method)	Women volunteers living with HIV Program working groups	10 7	✓ Yes C/I	✓	✓ Yes C/I	✓ Yes C/I	✓ Yes C/I	✗	Not described
**Perngmark et al.**	Action Research with Codesign Qualitative	Semi‐structured interviews, participant observations, workshops, meetings	Family dyads	10		✓ Yes C/I	✓ Yes C/I	✓ Yes C/I	✓	✓ Yes C/I	Not described
**Remien et al.**	Community‐Based Participatory Research (CBPR) Qualitative	Meetings, interviews, focus groups	HIV‐positive clinic patients in South Africa Patient advocates	33 3	✗	✓ Yes C/I	✗	✓ Yes C/I	✓	✓ Yes C/I	Not described
**Silcock et al.**	Experience Based Codesign, Medical Research Council Guidelines (MRC) Qualitative	Interviews with stakeholders, trigger film, user and carer meeting, joint meeting, design meeting, working groups	People 65 years and older, family members, others supporting these people on a daily basis, carer	14 carer/older people at user/carer meeting and 8 or 9 at subsequent initial/design/final meetings	✓ Yes C/I	✓ Yes C/I	✓ Yes C/I	✓ Yes C/I	✓ Yes C/I	✓ Yes C/I	Not described

*Note:* ✗= No consumer or stakeholder involvement ✓= other stakeholder involvement only, consumers not involved✓Yes C/I = consumer involvement in this stage.

**Bolded** author names represent the original 15 studies included in the review.

##### 3.3.1.1 Type of Consumers Involved

Most of the studies reported the type and number of consumers involved in the design and development of the medication safety intervention or service, although the demographic details of the consumers such as age, gender and socioeconomic position of the consumers and other stakeholders were poorly reported. The number of consumers involved in the design and development of the medication safety intervention or service ranged from 4 to 949 (Table 2). The language used by authors to describe the types of consumers involved varied and included patients, families, carers, consumer advisory groups/panels, older people, community members, people living with chronic illnesses, patient representatives/advocates, patient support groups, family dyads, service users and ‘experts by experience’.

#### 3.3.2 When Consumers Have Been Involved in the Research Studies [15, 40, 41]

Most of the studies involved consumers in both the exploratory and development stages and only two studies [36, 49] described consumer involvement across all six stages of the study (Table 2).

#### 3.3.3 Stage 1: Engage

Eight studies [36, 43, 44, 48, 49, 51, 54, 57] reported establishing and maintaining meaningful relationships with consumers and other stakeholders at the start of the project to understand and improve health care interventions or services [40, 41]. For example, one strategy described to enact this stage is to establish a patient advisory group [49] or a patient advisory panel [48]. The remaining studies did not describe establishing and maintaining meaningful relationships with consumers or other stakeholders before the start of the project.

#### 3.3.4 Stage 2: Plan

Eight studies [36, 44, 48, 49, 51, 55, 56, 58] described working with consumers and stakeholders to identify and generate ideas about the study goals and plans how to achieve them [40, 41]. One study planned the project with other stakeholders and did not appear to include consumers [54] and six studies did not describe consumer or stakeholder involvement in the planning stage of the study [43, 46, 47, 50, 52, 53].

#### 3.3.5 Stage 3: Explore

Twelve studies [36, 43, 47, 49, 50, 51, 53, 54, 56, 59, 60, 61] reported on exploring consumers experiences and identifying challenges, barriers, facilitators and ideas for medication safety interventions or services [40, 41]. Two studies did not include consumers in this stage and explored experiences with support staff, service managers and trainers [45] while another study explored barriers with health care practitioners [52]. One study did not describe an ‘explore’ stage as they adapted an existing intervention and focused on the improvements through the development stage rather than through the exploration stage of the design process [55]. Most studies undertook interviews, surveys/questionnaires, direct observation or focus groups to explore consumer perspectives and ideas.

#### 3.3.6 Stage 4: Develop

All of the studies included a ‘develop’ stage of the intervention or service where ideas from the consumers and/or stakeholders were turned into potential solutions or improvements [40, 41]. While 14 studies described including consumers in this stage, one study did not describe consumer involvement as findings from qualitative interviews were fed directly into design workshops and finalisation of the intervention [50]. Methods commonly used in the development stage included focus groups, workshops, codesign events and meetings.

#### 3.3.7 Stage 5: Decide

Eight studies [36, 45, 46, 49, 51, 53, 54, 59] described working with consumers to prioritise ideas for medication safety interventions or services and design intervention prototypes [40, 41]. The remaining studies did not describe consumer involvement in this stage but the involvement of other stakeholders.

#### 3.3.8 Stage 6: Change

The final stage was ‘change’ whereby pretesting of the prototype was undertaken with end users to gather further feedback and often to assess the acceptability, feasibility and usability of the intervention or service [40, 41]. Eight studies described involving consumers [36, 43, 44, 46, 47, 49, 55, 56]. Three studies involved other stakeholders in the ‘change’ stage but did not describe involving consumers [45, 48, 51] and four studies did not describe prototype testing with consumers or other stakeholders [50, 52, 53, 54].

### 3.4 Consumer Experience of Being Involved in the Design and Development of the Medication Safety Intervention or Service

Only one study across two articles [46, 62] explored the consumer experience of being involved in the research process through a questionnaire and semi‐structured interview. Respondents described a positive experience, stating that the codesign process used in the study was inclusive of their perspectives, facilitated learning by sharing experiences and built an environment that was inviting, creative and tolerant.

### 3.5 Evaluation of Medication Safety Intervention or Service

Intervention or service effectiveness was evaluated in nine of the studies and often described as feasibility, acceptability, efficacy, pilot testing or field testing (Table 1) [44, 45, 48, 55, 56, 63, 64, 65, 66]. Ben‐Zeev et al [63] found that whilst a mobile health intervention was helpful and acceptable to patients in reducing positive symptoms of psychopathology and depression there was no significant change in beliefs about medications. Mullan et al. [64] found that while their decision aid was acceptable to patients, it did not improve adherence or HbA1c levels. Cedillo et al. [44] showed that primary care providers were more adherent to treatment guidelines but there was no evidence of change in any patient‐centred measures like medication use and adherence. Deb et al.'s [45] SEPCTROM intervention's field testing showed it was acceptable, applicable, practical and relevant and improved staff knowledge of psychotropic medications and support to people with intellectual disabilities. Lee et al. [65] found that their clinical pharmacy model was well accepted by clients and carers and led to the resolution of identified medicine‐related problems hence contributing to enhanced medication safety. Kassavou et al. [66] found that a highly tailored digital intervention [47] was appealing to patients and effective at improving medication adherence. Latif et al. [48] found that a patient‐professional co‐produced digital education intervention to improve home medicine review referrals had no impact on the number of patients being offered and completing a medication review. Perngmark et al. [56] found that a program to promote medication adherence among older adults with uncontrolled hypertension improved medication adherence and blood pressure control. Remien et al. [55] reported that their adherence intervention was acceptable and feasible to patients but did not describe its effectiveness on medication adherence. Other studies, particularly those undertaken in the last few years did not describe any evaluation of the intervention or service but reported that future evaluation studies would be undertaken.

## 4 Discussion

Various consumer involvement approaches were identified in the studies in this review, with a wide range of differences in the types and numbers of consumers involved. We found a few of the studies in the review interchangeably used terms like codesign, coproduction and cocreation when describing consumer involvement within their study. This may have been because consumers had different levels of participation, or it may have been because of a lack of understanding about the differences in the approaches leading to inappropriate use of terminology. These terms are not the same and have distinct origins and features [67]. Some studies provided a detailed description of the consumer and community involvement approach whereas other studies referenced this to another article or gave a brief description, making it difficult to understand the nuances of consumer involvement in the work. Generally, we found studies describing consumer involvement approaches and the intervention or service design and development in a single article often lacked sufficient detail. This may have been because of word limit restrictions when publishing the reference, limiting the ability to describe consumer involvement in any meaningful depth. Of the articles that described consumer involvement, we found there was an inconsistent approach to reporting including terminology and detail, making it difficult for future researchers to replicate and compare. This is a finding that has been expressed in other reviews [37, 68]. As a post hoc observation, none of the studies reported following the GRIPP2 (Guidance for Reporting Involvement of Patients and the Public) reporting checklists [69] for patient and public involvement in research, which was designed to enhance the quality, transparency and consistency of patient and public involvement in research. These guidelines are helpful in providing consistency in language and reporting of consumer involvement processes, stages of involvement, level and nature of involvement, outcomes, impacts and outputs [69]. Furthermore, it is noted that none of the studies included in this review appeared to include consumers as coauthors of their papers.

Consumer recruitment occurred in different ways and most of the studies reported the type and number of consumers involved in the design and development of the medication safety intervention or service. Other characteristics of the consumers involved in the study were not well reported which made it difficult to establish if a diverse range of people had been involved throughout the study. One of the principles of consumer involvement and engagement is diversity and it is recommended that we try to involve people with a diverse range of skills and knowledge including those with lived experience, those who deliver or implement an intervention or service and other key stakeholders [26, 70]. Hatch et al. argue that attracting and supporting people from different demographic groups is important to ensure that all parts of society are empowered, represented and their voices heard in decisions that may affect their health and quality of life [71].

We were interested to see what methods or activities of engagement researchers had used to explore and understand consumer experiences and to generate ideas, to help us understand the most effective ways of engaging with consumers. While the most used methods in the studies aligned with those suggested by consumer involvement toolkits [41, 72], it was unclear as to whether consumers were involved in choosing the methods used in the studies to ensure that they were easy, flexible, acceptable and appropriate for all patients or consumers and that they created a safe and inclusive environment for people to share their experiences [26]. This is potentially important as it may have an impact on the recruitment of consumers to research studies. Some more creative approaches that were adopted to explore and generate consumers ideas and preferences were photovoice [54], public deliberation sessions [61], collective intelligence workshops [52] and scenario‐based design [52], but often there was insufficient detail reported to establish what was actually involved, making it difficult for future researchers to implement.

We used Boyd et al's codesign framework [40, 41] to determine which stages of the studies involved consumers. This was quite difficult to determine from the 15 included studies as several studies had multiple articles published separately describing different stages of the project. Consumers were least involved in the ‘plan’ and ‘engage’ stages of the project. Some authors argue that this is an important stage to involve consumers to minimise research waste [68], develop relationships [40], provide advice and contribute to the development of the methodology for the research project, grant and ethics applications, consent forms, plain language information about the project [18, 73] and discuss ideas for involvement activities [73]. Engaging consumers early means that their experiences and preferences can be taken into account at the start of the project and helps to ensure that researchers' priorities align with those of patients and service users and no assumptions are made about what is required [15, 40, 70]. The other stages where consumer involvement varied were in the ‘decide’ and ‘change’ stages. Approximately half of the studies did not describe consumer involvement in making final decisions about the medication safety intervention or service and the design of a prototype. Similarly, consumers were involved in approximately half of the intervention or service pretesting or testing of prototypes to gather further feedback and to assess usability and acceptability. Some authors recommend consumers be involved in all stages of research from defining the need/priority of research questions, refining the research questions and research design through to the conduct of the research and the dissemination of results [70, 73], while others suggest consumer involvement may vary depending on the project, resources and timelines [41, 72].

In addition to establishing how consumer involvement was implemented across the design and development of medication safety interventions or services it is important to examine the outcomes and impacts that consumer involvement has had on the research, the individuals involved and the wider impacts, including the positive and negative aspects. However, the lack of reporting of consumer involvement evaluation seems to be a common sentiment expressed by other authors [20, 68]. Reporting of consumer experience in the research process is valuable in that it provides guidance on what has worked well, was genuine, and meaningful and what can be improved in future studies involving consumers. This is important because consumer involvement can sometimes be viewed as tokenistic, particularly if decisions about the project are made in advance by researchers and other stakeholders and consumers are asked to purely provide feedback or if suggestions were not implemented [13]. It also provides an understanding of whether the designed intervention or service is a reflection of their needs and preferences [74].

It is unclear if consumers were involved in deciding on the evaluation measures or outcomes that were used in the studies, which may be important to ensure they are easy to use and appropriate for consumers to complete. Nevertheless, involving consumers appears to have a mixed influence on measurable clinical outcomes or effectiveness, and/or patient‐reported experience/outcome measures. Understanding the reasons for these mixed findings is beyond the scope of the current review, and also difficult to gauge because some studies did not set out to explore or report these outcomes. This is complicated by the inconsistent detail of reporting consumer involvement, and the multifaceted reasons for why interventions or services may or may not show effectiveness or improved outcomes, even with or without consumer involvement in the research processes.

## 5 Strengths and Limitations

A major strength of this review is that we opted to use a broad definition of consumer involvement to ensure that we included studies that had involved consumers across the research process but hadn't formally called this ‘codesign’ or one of the other approaches used to involve consumers. The reporting of consumer involvement within research papers is often inconsistent and lacking in detail, this limits the ability to understand the quality of the consumer involvement and how it worked and what worked well. More specifically, the reporting of core consumer stakeholder principles like trust, respect, equitable power between all research team members, inclusiveness, capacity building, advocacy and promoting shared and collaborative decision‐making [75] were unable to be discussed in depth because only one paper reported on consumer experience with the research process. We acknowledge that we did not include a consumer as a coauthor of our review, because this was undertaken as part of a PhD project, and we did not have resources to remunerate consumers for their time in this work. This is a limitation because consumers may have potentially brought a different lens, particularly around data extraction.

## 6 Conclusion

The benefit of involving consumers in medication safety interventions or services design and development has potentially not been maximised, given genuine consumer involvement across all stages of the research process appears to be the exception and not the norm. There is a need to improve quality, transparency and consistency of reporting [69], particularly around the description of the consumers involved, their experience of being involved, and overall impact and quality of participation, to better understand the impact of consumer involvement on outcomes and outputs of the research. Regardless of whether consumer involvement makes a difference to clinical outcomes, there is a moral imperative for consumers to be involved in research decisions that may impact their health and well‐being and that of their family and the broader community.

## Author Contributions

**Megan DelDot:** writing–original draft; conceptualisation; investigation; methodology; writing–review & editing; formal analysis. **Esther Lau:** investigation; methodology; writing–review & editing; formal analysis; conceptualisation; writing–original draft. **Nicole Rayner:** methodology; writing–review & editing; writing–original draft. **Jean Spinks:** writing–review & editing; conceptualisation. **Fiona Kelly:** writing–review & editing; conceptualisation. **Lisa Nissen:** writing–review & editing; conceptualisation.

## Conflicts of Interest

The authors declare no conflicts of interest.

## Data Availability

No new data were generated or analysed in support of this research.

## Appendix 1 Search Strategy

**Search Strategy Medline (EBSCOhost), 1946 to Present.** Table A1

**Table A1:** Base search strategy.

Search No.	Query
S01	TI ((consumer* OR patient* OR carer* OR caretaker* OR caregiver* OR stakeholder* OR famil* OR “enduser*“ OR user* OR “multi stakeholder*“ OR multistakeholder) N10 (“codesign*“ OR codesign* OR coproduc* OR “co‐produc*“ OR cocreat* OR “co‐creat*“ OR “co‐owner*“ OR “co‐owner*“ OR “co‐evaluat*“ OR “coevaluat*“ OR “co‐decide*“ OR “codecide*“ OR “codecision*“ OR “co‐decision*“ OR “coinvolve*“ OR “co‐involve*“ OR “copartner*“ OR “copartner*“)) OR AB ((consumer* OR patient* OR carer* OR caretaker* OR caregiver* OR stakeholder* OR famil* OR “enduser*“ OR user* OR “multi stakeholder*“ OR multistakeholder) N10 (“codesign*“ OR codesign* OR coproduc* OR “co‐produc*“ OR cocreat* OR “co‐creat*“ OR “co‐owner*“ OR “co‐owner*“ OR “co‐evaluat*“ OR “coevaluat*“ OR “co‐decide*“ OR “codecide*“ OR “codecision*“ OR “co‐decision*“ OR “coinvolve*“ OR “co‐involve*“ OR “copartner*“ OR “copartner*“))
S02	TI “trigger film*“ OR AB “trigger film*“
S03	TI ((consumer* OR “experience based”) AND (“co design*“ OR codesign* OR “co produc*“ OR coproduc* OR “co creat*“ OR cocreat*)) OR AB ((consumer* OR “experience based”) AND (“co design*“ OR codesign* OR “co produc*“ OR coproduc* OR “co creat*“ OR cocreat*))
S04	TI (consumer* N2 (engage* OR involv* OR feedback OR leader* OR participa* OR champion* OR empower* OR led OR driven OR particip* OR design* OR creat* OR produc* OR panel* OR partner*)) OR AB (consumer* N2 (engage* OR involv* OR feedback OR leader* OR participa* OR champion* OR empower* OR led OR driven OR particip* OR design* OR creat* OR produc* OR panel* OR partner*))
S05	TI (patient* N2 collaborat*) OR AB (patient* N2 collaborat*)
S06	TI (“participatory action*“ OR “formative design*“ OR “codesign*“ OR “co design*“) OR AB (“participatory action*“ OR “formative design*“ OR “codesign*“ OR “co design*“)
S07	TI ((“patient reported” OR “experience based”) N2 (“co design*“ OR codesign* OR measure OR experience*)) OR AB ((“patient reported” OR “experience based”) N2 (“co design*“ OR codesign* OR measure OR experience*))
S08	TI (community N2 (“codesign*“ OR codesign* OR coproduc* OR “co‐produc*“ OR cocreat* OR “co‐creat*“ OR “co‐owner*“ OR “co‐owner*“ OR “co‐evaluat*“ OR “coevaluat*“ OR “co‐decide*“ OR “codecide*“ OR “codecision*“ OR “co‐decision*“ OR “coinvolve*“ OR “co‐involve*“ OR “copartner*“ OR “copartner*“ OR “participa*“)) OR AB (community N2 (“codesign*“ OR codesign* OR coproduc* OR “co‐produc*“ OR cocreat* OR “co‐creat*“ OR “co‐owner*“ OR “co‐owner*“ OR “co‐evaluat*“ OR “coevaluat*“ OR “co‐decide*“ OR “codecide*“ OR “codecision*“ OR “co‐decision*“ OR “coinvolve*“ OR “co‐involve*“ OR “copartner*“ OR “copartner*“ OR “participa*“))
S09	(MH “Community Participation + “) OR (MH “Community‐Based Participatory Research + “)
S10	S1 OR S2 OR S3 OR S4 OR S5 OR S6 OR S7 OR S8 OR S9
S11	TI ((medication* OR medicine* OR drug$ OR patient*) N5 (“safe* use” OR safet* OR harm OR error* OR “safe* medic*“)) OR AB ((medication* OR medicine* OR drug$ OR patient*) N5 (“safe* use” OR safet* OR harm OR error* OR “safe* medic*“))
S12	TI (deprescri* OR overprescri* OR “pharmaceutical prep*“ OR “prescri* error*“) OR AB (deprescri* OR overprescri* OR “pharmaceutical prep*“ OR “prescri* error*“)
S13	TI ((medication* OR medicine*) N3 (therapies OR therapy OR manage* OR “review*“ OR “optimi*ation” OR “adhere*“ OR “nonadhere*“ OR comply* OR compli* OR noncomply* OR noncompli* OR “non compli*“ OR “non comply*“ OR “non adhere*“)) OR AB ((medication* OR medicine*) N3 (therapies OR therapy OR manage* OR “review*“ OR “optimi*ation” OR “adhere*“ OR “nonadhere*“ OR comply* OR compli* OR noncomply* OR noncompli* OR “non compli*“ OR “non comply*“ OR “non adhere*“))
S14	(MH “Medication Therapy Management”) OR (MH “Patient Safety”) OR (MH “Patient Harm”) OR (MH “Medication Errors + “) OR (MH “Consumer Product Safety”) OR (MH “Potentially Inappropriate Medication List”) OR (MH “Medication Adherence + “) OR (MH “Medication Reconciliation”) OR (MH “Pharmaceutical Preparations”)
S15	S11 OR S12 OR S13 OR S14
S16	TI ((service OR intervention* OR program*) N5 (plan* OR safety OR program* OR delivery OR improv* OR intergrat*)) OR AB ((service OR intervention* OR program*) N5 (plan* OR safety OR program* OR delivery OR improv* OR intergrat*))
S17	TI ((user* OR patient* OR consumer* OR human) N2 (centred OR centred OR “decision*“ OR “shared”)) OR AB ((user* OR patient* OR consumer* OR human) N2 (centred OR centred OR “decision*“ OR “shared”))
S18	TI (dashboard* OR tool$ OR toolkit* OR intervention OR “decision aid”) OR AB (dashboard* OR tool$ OR toolkit* OR intervention OR “decision aid”)
S19	TI (“practice guide*“ OR consensus OR “clinical guide*“) OR AB (“practice guide*“ OR consensus OR “clinical guide*“)
S20	(MH “Health Planning + “) OR (MH “Practice Guidelines as Topic”) OR (MH “Decision Making, Shared”) OR (MH “Consensus”)
S21	S16 OR S17 OR S18 OR S19 OR S20
S22	S10 AND S15 AND S21
S23	((MH “Infant + “) OR (MH “Child + “) OR (MH “Adolescent”)) NOT (MH “Adult + “)
S24	S22 NOT S23

## Appendix 2

Table A2

**Table A2:** Data extraction form.
